# Gasdermin E regulates the stability and activation of EGFR in human non-small cell lung cancer cells

**DOI:** 10.1186/s12964-023-01083-7

**Published:** 2023-04-21

**Authors:** Limei Xu, Feifei Shi, Yingdi Wu, Shun Yao, Yingying Wang, Xukai Jiang, Ling Su, Xiangguo Liu

**Affiliations:** 1 Shandong Provincial Key Laboratory of Animal Cell and Developmental Biology, School of Life Sciences, Shandong University, Room N8-108, 72 Binhai Road, Qingdao, 266237 People’s Republic of China; 2 National Glycoengineering Research Center, Shandong University, Qingdao, China

**Keywords:** EGFR, GSDME, ERK1/2, Proliferation

## Abstract

**Background:**

Lung cancer is the most lethal malignancy, with non-small cell lung cancer (NSCLC) being the most common type (~ 85%). Abnormal activation of epidermal growth factor receptor (EGFR) promotes the development of NSCLC. Chemoresistance to tyrosine kinase inhibitors, which is elicited by EGFR mutations, is a key challenge for NSCLC treatment. Therefore, more thorough understanding of EGFR expression and dynamics are needed.

**Methods:**

Human non-small cell lung cancer cells and HEK293FT cells were used to investigate the molecular mechanism of gasdermin E (GSDME) regulating EGFR stability by Western blot analysis, immunoprecipitation and immunofluorescence. GSDME and EGFR siRNAs or overexpression plasmids were used to characterize the functional role of GSDME and EGFR in vitro. EdU incorporation, CCK-8 and colony formation assays were used to determine the proliferation ability of non-small cell lung cancer cells.

**Results:**

GSDME depletion reduced the proliferation of non-small cell lung cancer cells in vitro. Importantly, both GSDME-full length (GSDME-FL) and GSDME-N fragment physically interacted with EGFR. GSDME interacted with cytoplasmic fragment of EGFR. GSDME knockdown inhibited EGFR dimerization and phosphorylation at tyrosine 1173 (EGFR^Y1173^), which activated ERK1/2. GSDME knockdown also promoted phosphorylation of EGFR at tyrosine 1045 (EGFR^Y1045^) and its degradation.

**Conclusion:**

These results indicate that GSDME-FL increases the stability of EGFR, while the GSDME N-terminal fragment induces EGFR degradation. The GSDME-EGFR interaction plays an important role in non-small cell lung cancer development, reveal a previously unrecognized link between GSDME and EGFR stability and offer new insight into cancer pathogenesis.

**Video abstract**

**Supplementary Information:**

The online version contains supplementary material available at 10.1186/s12964-023-01083-7.

## Background

Lung cancer is the most lethal malignancy worldwide [1], and approximately 85% of lung cancers are non-small cell lung cancer (NSCLC). NSCLC is subdivided into lung squamous cell carcinoma (LUSC, ~ 30%), lung adenocarcinoma (LUAD, ~ 50%) and other types (~ 20%) [2]. Epidermal growth factor receptor (EGFR, also known as ERBB1) belongs to the ERBB family of transmembrane receptor tyrosine kinases (RTKs) [3, 4]. Under physiological conditions, EGFR is in an autoinhibited, dimerization-incompetent state at the plasma membrane. Ligand binding promotes receptor dimerization, leading to the allosteric activation of EGFR kinase and to the trans-autophosphorylation of critical tyrosine residues in the cytoplasmic receptor tail, therefore triggering intracellular signaling cascades, such as the PI3K/AKT and RAS/RAF/MEK/ERK pathways, which are critical for cell proliferation, survival, motility and differentiation [3, 5–7]. Once activated at the plasma membrane, EGFR is rapidly ubiquitinated by the E3 ligase c-Cbl and internalized to endosomes then eventually being degraded in lysosome [3, 7, 8]. The proteasomal pathway is also involved in EGFR degradation [9]. Dysregulation of EGFR protein stability contributes to abnormal EGFR signaling and cancer development [3, 10]. Therefore, it is fundamentally important to elucidate the molecular mechanisms by which EGFR protein stability is controlled. Many studies have shown that EGFR is overexpressed in a variety of cancers, including lung, glioma, esophageal, colorectal and head and neck cancer [5, 6, 11]. EGFR is also overexpressed in NSCLC [12], and activation of EGFR signaling (EGFR gene amplification and active mutations) is a critical driving force for NSCLC (~ 85%) [10]. Among the emerging targeted therapies, EGFR tyrosine kinase inhibitors (TKIs, such as gefitinib and erlotinib) have been widely used for the treatment of EGFR-positive NSCLC patients [13]. Although remarkable clinical efficacy has been demonstrated in the treatment of patients with EGFR mutations the past decade, the majority of NSCLC patients acquire resistance to EGFR-TKIs, and new acquired mutations related to EGFR inhibitor treatment have continuously emerged [14]. Therefore, novel strategies to combat TKI-resistant NSCLC are urgently needed to overcome drug resistance.

Gasdermin E (GSDME) was originally identified as deafness, autosomal dominant 5 (DFNA5), and genetic mutations of DFNA5 cause nonsyndromic deafness in humans [15]. GSDME belongs to a gasdermin family (including GSDMA, GSDMB, GSDMC, GSDMD, DFNA5 and DFNB59) that shares the pore-forming domain (except for DFNB59) [16]. GSDME was recently identified as an executor of pyroptosis (an inflammatory form of programmed cell death executed by the gasdermin family of pore-forming proteins) owing to its cleavage by caspase-3 [17]. Caspase-3 cleaves GSDME in its middle linker to release autoinhibition on its gasdermin-N domain, resulting in the generation of an ~ 35 kDa fragment, which permeabilizes the plasma membrane via its pore-forming activity. Under physiological conditions, GSDME adopts a two-domain architecture (GSDME-N and GSDME-C), and the two domains are capable of binding to each other and autoinhibition. The GSDME-N domain but not the full-length protein can induce mammalian cell pyroptosis [16].

Aberrant gene expression is a characteristic of cancer, and changes in DNA methylation status have profound effects on gene expression. GSDME was identified in several screens for genes methylated in gastric cancer [18], colorectal cancer [19] and breast cancer [20, 21]. It was also reported that GSDME suppressed tumor growth by promoting the antitumor functions of tumor-infiltrating NK and CD8^+^ T killer lymphocytes [22]. These studies suggested GSDME as a putative tumor suppressor in various types of cancer. However, a recent study revealed that the protein levels of GSDME were significantly increased in the colonic mucosa of inflammatory bowel disease (IBD) patients compared to healthy controls and that GSDME increased release of HMGB1 (one of the DAMPs that has been recognized) from intestinal epithelial cells (IECs), which contributed to colitis-associated cancer (CAC) tumorigenesis [23], suggesting that GSDME might exert dual roles in tumor progression.

In the present study, we demonstrated a critical role of GSDME in the maintenance of EGFR stability. We found that GSDME depletion reduced the proliferation of NSCLC cancer cells in vitro. Importantly, both GSDME-FL and the GSDME-N fragment physically interacted with EGFR. GSDME interacted with the cytoplasmic fragment (CT) of EGFR. GSDME knockdown inhibited EGFR dimerization and phosphorylation at tyrosine 1173 (EGFR^Y1173^), which activated ERK1/2. GSDME knockdown also promoted phosphorylation of EGFR at tyrosine 1045 (EGFR^Y1045^) and its degradation. Moreover, GSDME-FL increased the stability of EGFR, while the GSDME-N fragment induced EGFR degradation. Together, our results demonstrate that the GSDME-EGFR interaction plays an important role in the progression of EGFR-positive NSCLC cells.

## Materials and methods

### Reagents and antibodies

Doxorubicin and MG132 (S2619) were purchased from Selleck (China). EGF was purchased from Sangon Biotech (Shanghai, China). E64D was from Medchem Express. CHX (T1225), a eukaryotic protein synthesis inhibitor, was purchased from TOPSCIENCE (China). The EdU cell proliferation assay kit was from Beyotime (China). CCK-8 was purchased from Vazyme (Vazyme Biotech Co., Ltd.). Antibodies against GSDME (84005), phospho-ERK1/2 (4370), ERK1/2 (9102) and CCND1 (2926) were purchased from Cell Signaling Technology. GSDME (84005) antibody recognizes both endogenous levels of total GSDME protein and the GSDME N-terminal fragment. Anti-EGFR (sc-03) was acquired from Santa Cruz Biotechnology. Antibodies against phospho-EGFR (Y1045, PA5-17816) and phospho-EGFR (Y1173, MA5-15158) were purchased from Invitrogen. Anti-ACTB (A1978), anti-GAPDH (G8795), anti-FLAG (F1804-M; F7425-R), anti-HIS (SAB1306085), and anti-EEA1 (E7659) were purchased from Sigma‒Aldrich. Anti-HA (66006–1-IG) and anti-c-Cbl (25818-1-AP) were purchased from Proteintech. Alexa Fluor 488 (M) and Alexa Fluor 555 (R) were purchased from Invitrogen.

### Cell culture

The A549, Calu-1, H157, H1299 and H1792 cell lines were cultured in RPMI 1640 medium (Sigma‒Aldrich) containing 10% fetal bovine serum (FBS). A549, Calu-1, H1299 and H1792 were lung adenocarcinoma (LUAD) cell lines, H157 was lung squamous cell carcinoma (LUSC) cell line. HEK293FT cells were cultured in DMEM (Sigma‒Aldrich) supplemented with 10% FBS. All medium contained 100 units/mL penicillin and 100 µg/mL streptomycin. All cell lines were originally obtained from the American Type Culture Collection (ATCC) and were cultured in a humidified atmosphere of 5% CO_2_ at 37 °C.

### RNA interference

Small interfering RNAs (siRNAs) were synthesized by GenePharma (Shanghai, China). Scrambled siRNA (siScr) targeted the sequence 5′-TTCTCCGAACGTGTCACGT-3′. GSDME siRNA-1 (siGSDME-1) and GSDME siRNA-2 (siGSDME-2) target the sequences 5′-TGATGGAGTATCTGATCTT-3′ and 5′-GTCATCTGTGAAAGCTGTC-3′, respectively. EGFR siRNA-1 (siEGFR-1) and EGFR siRNA-2 (siEGFR-2) target the sequences 5′-CCTTAGCAGTCTTATCTAATT-3′ and 5′-TACGAATATTAAACACTTCTT-3′, respectively. The siRNA targeting human GSDME or EGFR was applied as a mixture at a total final concentration of 50 nM. Cells were transfected with siRNAs using jetPRIME transfection reagent (Polyplus) according to the manufacturer’s instructions.

### Plasmid construction and transfection

The pcDNA3.1 plasmid was purchased from Invitrogen (V79020). The GSDME ORF sequence was amplified from the cDNA of HEK293FT cells using the following primers: sense, 5′- GGGATCCGCCGCCACCATGGACTACAAAGACGATGACGACAAGTTTGCCAAAGCAACCAGG -3′ and antisense, 5′- CCTCGAGTCAGGCATAATCGGGTACATCGTAAGGGTATGAATGTTCTCTGCCTAAAGC -3′. The EGFR gene with a FLAG tag was amplified from the cDNA of H1299 cells using the following primers: sense, 5′-CGGTACCGCCGCCACCATGCGACCCTCCGGGACGGCCGGGGC-3′ and antisense, 5′-CCTCGAGTCACTTGTCGTCATCGTCTTTGTAGTCTGAAATTCACTGCT-3′. All the plasmids used were cloned into pcDNA3.1 and confirmed by DNA sequencing. Plasmids were transfected with LipoMax transfection reagent (Sudgen Biotechnology) in serum-free Opti-MEM (Gibco) according to the manufacturer’s instructions.

### EdU proliferation assay

Cells transfected with scrambled siRNA or siRNA targeting GSDME were plated in 6-well plates and incubated for 24 h. Cell proliferation was detected using the incorporation of 5-ethynyl-2′-deoxyuridine (EdU) with an EdU cell proliferation assay kit (Beyotime, China) according to the manufacturer’s instructions. Briefly, the cells were incubated with 10 μM EdU for 2 h. Next, cells were collected and fixed with 1 mL Immunol staining fix solution for 15 min at room temperature, washed with 1 mL Immunol staining blocking buffer three times, and incubated with 500 μL Click reaction buffer staining for 30 min at room temperature. The cells were washed 3 times with 1 mL Immunol staining blocking buffer, and the proportion of EdU-positive cells was determined by flow cytometry (Guava® easyCyte™, Merck Millipore) using 488 nm excitation. A total of 20,000 viable cells were analyzed. The samples were prepared in triplicate, and the data were presented using FlowJo software (Additional file 1: Fig. S7).

### Clonogenic assay

Cells transfected with scrambled siRNA or siRNA targeting GSDME for 24 h were replated in 6-well culture plates at a density of 400 cells/mL. Then, the cells were cultured in a humidified atmosphere of 5% CO_2_ at 37 °C for 14 days. Finally, the medium was carefully removed by aspiration, and the cells were washed with PBS three times. Then, the cells were fixed with 1 mL methanol for 15 min at room temperature. Colonies were stained with 0.05% crystal violet for 30 min at room temperature, photographed, and counted. Colonies of more than 50 cells were counted. The samples were prepared in triplicate.

### Cell proliferation

A CCK-8 assay was used to examine cell viability. In brief, cells transfected with scrambled siRNA or siRNA targeting GSDME for 24 h were reseeded in 96-well culture plates at 3 × 10^3^ cells/100 μL. Then, the cells were cultured for the indicated times in a humidified atmosphere of 5% CO_2_ at 37 °C. Finally, 10 μL of CCK-8 reagent was added to each well and incubated for 2 h at 37 °C. The absorbance of each well was measured at 450 nm. The samples were prepared in triplicate.

### Cell cycle analysis

Cells were transfected with scrambled siRNA (siScr) or siRNA targeting GSDME (siGSDME) for 48 h. Cell cycle distribution was determined using cell cycle and apoptosis analysis kit (Beyotime, China) according to the manufacturer’s instructions. Briefly, cells were washed three times with PBS, trypsinized, and collected by centrifugation at 1000 rpm for 5 min. Then cells were fixed in 3 mL of cold 75% ethanol at − 20 °C overnight. After centrifugation at 1600 rpm for 10 min, cells were washed by 5 mL cold PBS and centrifugated at 1600 rpm for 10 min, cell pellets were resuspended and incubated with 0.5 mL of buffer containing 100 μg/mL RNase and 5 μg/mL propidium iodide at 37 °C for 30 min. Cell cycle distribution was determined by flow cytometry (Guava® easyCyte™, Merck Millipore) using 488 nm excitation. A total of 10,000 viable cells were analyzed. The samples were prepared in triplicate, and the data were presented using Guava® easyCyte™ software.

### Western blot analysis

Cells were harvested and lysed on ice for 30 min and then centrifuged at 4 °C for 15 min. Concentration of proteins were determined by bradford protein assay kit (Beyotime, China) according to the manufacturer’s instructions. Protein samples (20 μg per lane) were separated by SDS‒PAGE (5–15%), electrotransferred onto PVDF membranes, and then blocked with 5% nonfat milk for 1 h at room temperature. Subsequently, the membrane was incubated with specific primary antibodies overnight at 4 °C. Proteins of interest were detected with appropriate secondary antibodies for 1 h at room temperature and detected by an HRP system. The protein levels were normalized to those of β-Actin or GAPDH. Band intensities were quantified by Image J software.

### Immunoprecipitation

Cells were harvested and lysed in IP lysis buffer (20 mM Tris–HCl, pH 7.5; 1 mM EGTA; 150 mM NaCl; 1 mM Na_2_EDTA; 2.5 mM sodium pyrophosphate; 1 mM Na_3_VO4; 1 mM β-glycerophosphate; 1% Triton) supplemented with 1% protease inhibitors on ice for 30 min and then centrifuged at 4 °C for 15 min. The supernatants were incubated with the antibody at 4 °C for 1 h. Next, the mixture was incubated with protein A beads at 4 °C overnight. Beads were washed with 1 mL of lysis buffer three times, and the supernatants were removed. Immunoprecipitated proteins were then eluted using 20 μL 2 × SDS buffer (100 °C, 10 min). Samples were analyzed by Western blot analysis. 500 μg protein samples were separated by SDS‒PAGE (5–15%).

### Immunofluorescence

Cells transfected with scrambled siRNA or siRNA targeting GSDME were seeded on coverslips for the indicated time, washed with PBS three times and fixed in 4% paraformaldehyde for 15 min. Subsequently, the cells were permeabilized in 0.2% Triton X-100 for 10 min, blocked in 10% normal goat serum for 1 h at 4 °C and incubated with EEA1 (E7659, Sigma‒Aldrich) and EGFR (sc-03, Santa Cruz) antibodies overnight at 4 °C. The next day, following washing with PBS three times, Alexa Fluor 488 and Alexa Fluor 555 secondary antibodies were added to the sections and incubated for 1 h at room temperature. The sections were washed in PBS three times and counterstained with DAPI. Fluorescence images were visualized with a laser scanning confocal microscope.

### Molecular docking analysis

We performed protein‒protein docking for GSDME and the cytoplasmic domain of EGFR using the ClusPro 2.0 server [24]. The 3D structure of EGFR was obtained from the RCSB Protein Data Bank (PDB code: 4RJ4) [25]. The water and ligand molecules in the original crystal structure were removed. The 3D structure of GSDME was predicted through the AlphaFold2 method as described [26]. The complex of GSDME-EGFR with the best docking score was used to analyze the interaction between them. Chimera X was used to visualize the overall structure of proteins, and PyMOL (www.pymol.org) was used to analyze the hydrogen bonding interaction between both proteins [27, 28].

### Statistical analysis

The results of cell culture experiments were collected from at least three independent replicates. Data are presented as the means ± standard deviations (SDs). Statistical significance was calculated using unpaired Student’s *t* tests by GraphPad Prism 8.0. Statistical significance was also taken as *p < 0.05; **p < 0.01; ***p < 0.001; ****p < 0.0001; NS, not significant.

## Results

### GSDME depletion reduces proliferation of NSCLC cells in vitro

**Fig. 1:**
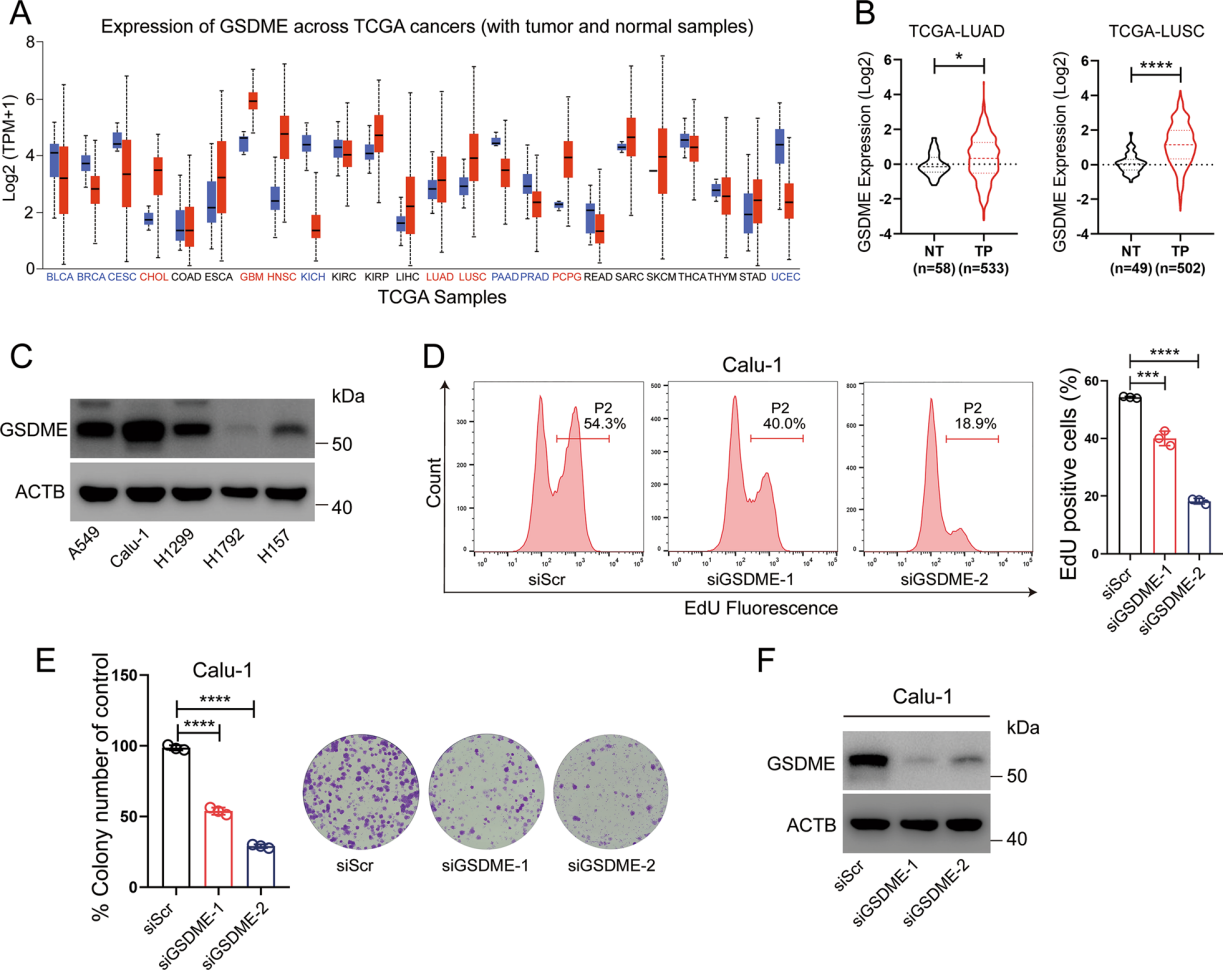
GSDME depletion suppresses the proliferation of NSCLC cells in vitro. **A** *GSDME* mRNA expression in TCGA cancer samples and normal samples from UALCAN. High expression of *GSDME* across TCGA cancers: CHOL, GBM, HNSC, LUAD, LUSC and PCPG (Red color). Low expression of *GSDME* across TCGA cancers: BLCA, BRCA, CESE, KICH, PAAD, PRAD and UCEC (Blue color). **B** Relative mRNA level of *GSDME* in TCGA LUAD samples (NT, *n* = 58; TP, *n* = 533; *p* < 0.05) and LUSC samples (NT, *n* = 49; TP, *n* = 502; *p* < 0.0001) compared with normal samples. **C** The protein levels of GSDME in A549, Calu-1, H1299, H1792 and H157 cells were measured by Western blot analysis. β-Actin was used as the loading control. **D** EdU incorporation measured by flow cytometry in Calu-1 cells transfected with siScr or siGSDME for 48 h. **E** Colony formation efficiency of Calu-1 cells after transfection with scrambled siRNA (siScr) or siRNA targeting GSDME (siGSDME) for 14 days. **F** The RNAi efficiency of Calu-1 cells was measured by Western blot analysis. β-Actin was used as the loading control. Data are presented as the mean ± SD and are representative of three independent experiments. The significant differences between the two groups were analyzed by two-sided unpaired Student’s *t test*s (*p < 0.05, ***p < 0.001, ****p < 0.0001)

GSDME is a newly recognized executor of pyroptosis and is usually silenced in various cancers as a putative tumor suppressor due to promoter hypermethylation [29, 30]. However, the expression level and molecular mechanisms of GSDME in NSCLC cells remain to be fully elucidated. Pancancer analysis of *GSDME* mRNA expression from UALCAN (http://ualcan.path.uab.edu/analysis.html) revealed that GSDME was upregulated obviously in several cancers, for example CHOL, GBM, HNSC and PCPG (Fig. 1A). We also explored the *GSDME* mRNA expression in the TCGA-LUAD/LUSC chorts, using the cBioPortal web service (http://www.cbioportal.org/). As shown in Fig. 1B, the *GSDME* mRNA level was upregulated in LUAD (NT, *n* = 58; TP, *n* = 533; *p* < 0.05) and LUSC samples (NT, *n* = 49; TP, *n* = 502; *p* < 0.0001) compared with normal samples. This result was consistent with that of a recent study [31]. Moreover, in contrast to previous observations that GSDME expression was negative or low within other cancer cells [18, 19], GSDME protein levels were examined by Western blot analysis in several NSCLC cell lines, including A549, Calu-1, H1299, H1792 and H157 cells. GSDME protein expression was detected in most of these cells (Fig. 1C). To further investigate whether GSDME suppresses the growth of human lung cancer cells, we knocked down GSDME by transfecting NSCLC cancer cells with GSDME-specific siRNA and evaluated cell proliferation. The EdU incorporation assay revealed a reduced rate of EdU incorporation in GSDME knockdown Calu-1, H1299 and A549 cells compared with control cells (Fig. 1D and Additional file 1: Fig. S1A and C). Colony formation and CCK-8 assays also showed that GSDME inhibition led to reduced proliferation (Fig. 1E, and Additional file 1: Fig. S1E-G). The RNAi efficiency of Calu-1, H299, A549 and H157 cells was measured by Western blot analysis (Fig. 1F and Additional file 1: Fig. S1 B, D and H). In summary, GSDME knockdown reduced the proliferation of NSCLC cells.

### GSDME silencing decreases the protein levels of CCND1 and phosphorylated ERK1/2 in NSCLC cells

**Fig. 2:**
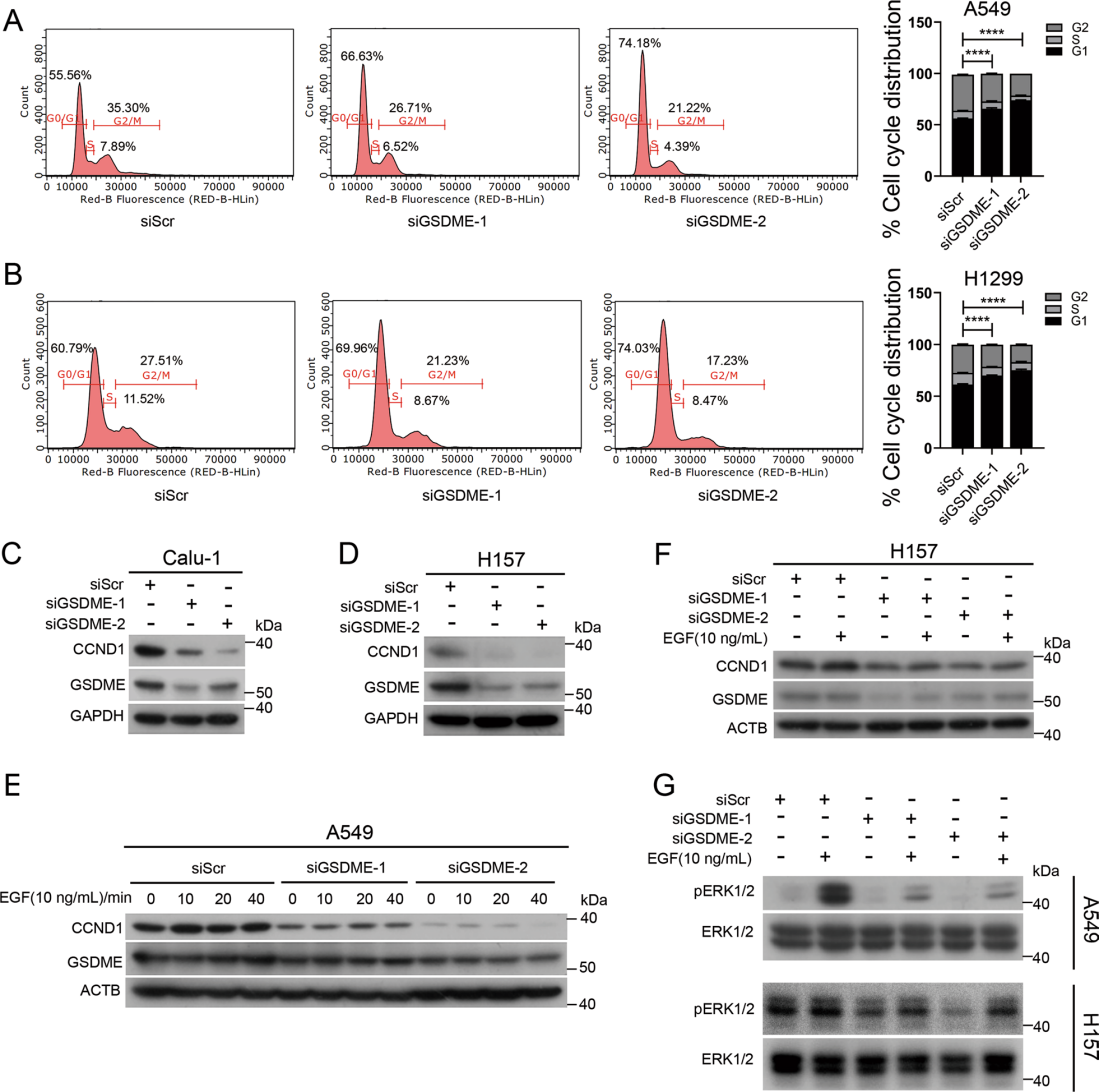
GSDME knockdown decreases the protein levels of CCND1 and phosphorylation of ERK1/2 in NSCLC cells. **A**, **B** Cell cycle distribution of A549 and H1299 cells after transfected siScr or siGSDME for 48 h. The cell cycle distribution was measured by flow cytometry.** C** Western blot analysis of CCND1 and GSDME levels in Calu-1 cells. **D** Western blot analysis of CCND1 and GSDME levels in H157 cells. GAPDH was used as the loading control. **E** Western blot analysis of CCND1 and GSDME levels in A549 cells treated with 10 ng/mL EGF for 0, 10, 20 and 40 min after transfection with siScr or siGSDME for 48 h. β-Actin was used as the loading control. **F** Western blot analysis of CCND1 and GSDME in H157 cells treated with 10 ng/mL EGF for 30 min after transfection with siScr or siGSDME for 48 h. β-Actin was used as the loading control **G** Western blot analysis of the phosphorylation of ERK1/2 in A549 and H157 cells treated with 10 ng/mL EGF for 30 min after transfection with siScr or siGSDME for 48 h. ERK1/2 were used as the loading control. Data are presented as the mean ± SD and are representative of three independent experiments. The significant differences between the two groups were analyzed by two-sided unpaired Student’s *t *tests (**** p < 0.0001)

We investigated whether GSDME knockdown exerted its proliferation inhibitory effect by regulating cell cycle. As shown in Fig. 2A, B, the cell cycle distribution in A549 and H1299 cells transfected with GSDME siRNA showed an increased accumulation of cells at G0/G1 phase. Then we examined the protein level of CCND1 (a key G1/S transition-related protein) by Western blot analysis in Calu-1, H157 and A549 cells. As shown in Fig. 2C–E, the protein level of CCND1 was notably decreased in GSDME knockdown cells. Because the ERK1/2 pathway (consisting of kinases RAS, RAF and ERK1/2) is known to activate the expression of CCND1 [32], and the hyperactivation of ERK1/2 drives cancer cell growth in various cancers [33, 34]. We next determined whether GSDME promotes the phosphorylation of ERK1/2 in A549 and H157 cells. As shown in Fig. 2G, the phosphorylation of ERK1/2 was decreased in GSDME knockdown cells. These results indicate that GSDME knockdown decreases the protein levels of CCND1 and the phosphorylation of ERK1/2 in NSCLC cells.

### GSDME physically interacts with EGFR

**Fig. 3:**
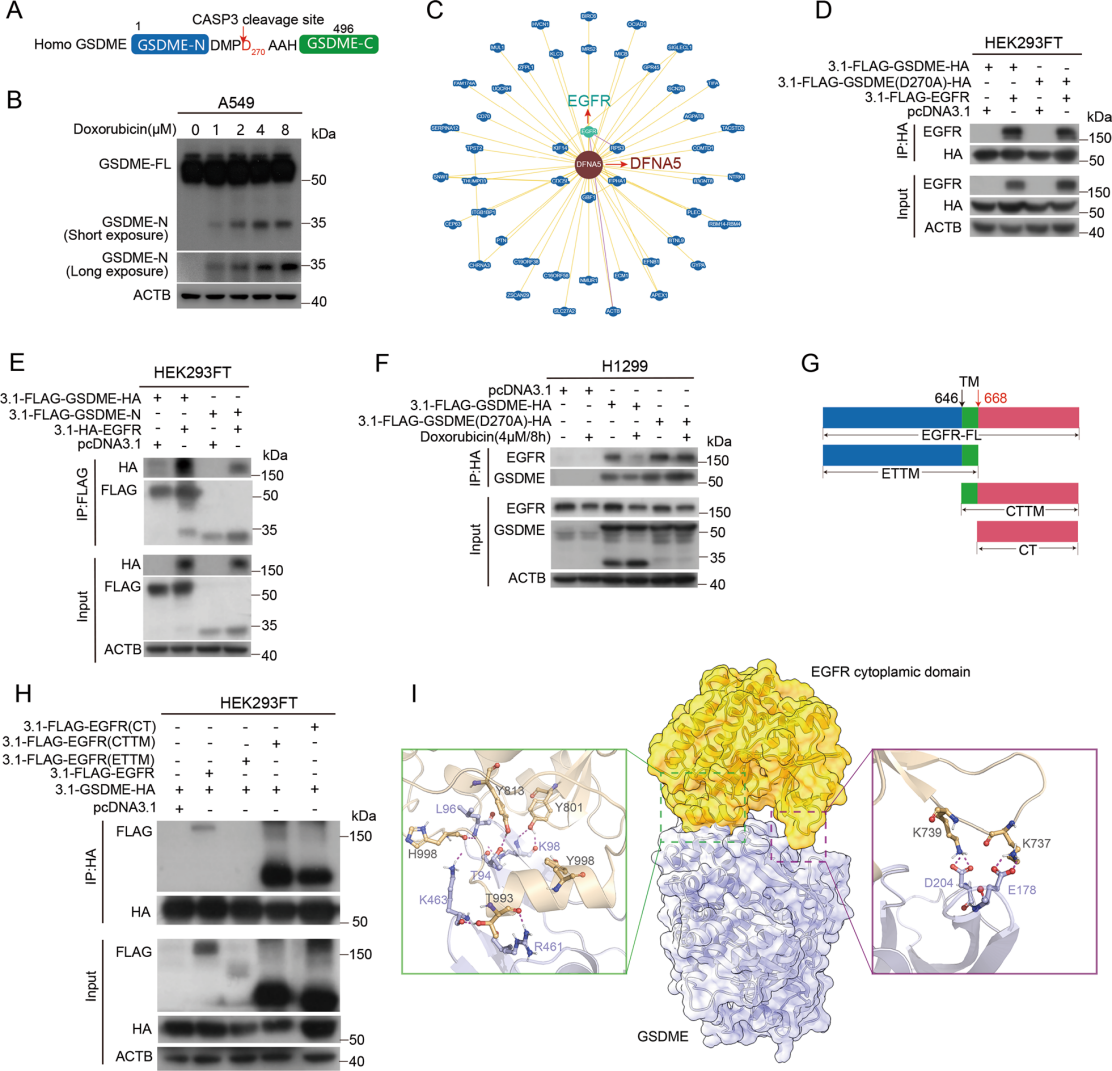
GSDME physically interacts with EGFR. **A** Diagram of the GSDME structure and the caspase-3 cleavage sites (Asp^270^). **B** A549 cells were treated with 0, 1, 2, 4 or 8 μM doxorubicin for 24 h, and GSDME-FL and GSDME-N were detected by Western blot analysis. GSDME-FL, full-length GSDME; GSDME-N, the N-terminal cleavage products of GSDME. **C** Interaction network of GSDME (also named DFNA5) analyzed in the BioGRID database. **D** HEK293FT cells were transfected with pcDNA3.1, pcDNA3.1-FLAG-EGFR, pcDNA3.1-FLAG-GSDME (D270A)-HA or pcDNA3.1-FLAG-GSDME-HA plasmids for 24 h. A co-IP assay was performed with HA antibody, and the coeluted proteins were detected by Western blot analysis with EGFR and HA antibodies. **E** HEK293FT cells were transfected with pcDNA3.1, pcDNA3.1-HA-EGFR, pcDNA3.1-FLAG-GSDME-N or pcDNA3.1-FLAG-GSDME-HA plasmids for 24 h. A Co-IP assay was performed with a FLAG antibody, and the coeluted proteins were detected by Western blot analysis with HA and FLAG antibodies. **F** H1299 cells were transfected with pcDNA3.1, pcDNA3.1-FLAG-GSDME-HA or pcDNA3.1-FLAG-GSDME (D270A)-HA plasmids for 24 h and treated with or without 4 μM doxorubicin for 8 h. A Co-IP assay was performed with HA antibody, and the coeluted proteins were detected by Western blot analysis with EGFR and GSDME antibodies. **G** Schematic diagram of EGFR. ETTM, extracellular and transmembrane fragment; CTTM, cytoplasmic and transmembrane fragment; CT, cytoplasmic fragment. **H** The interaction of GSDME with different fragments (CTTM, CT, ETTM) of EGFR was detected by a co-IP assay in HEK293FT cells. A co-IP assay was performed with HA antibody, and the coeluted proteins were detected by Western blot analysis with FLAG and HA antibodies. β-Actin was used as the loading control for the input. **I** Molecular docking analysis for GSDME and the cytoplasmic domain of EGFR using the ClusPro 2.0 server

When treated with chemotherapeutic agents (such as doxorubicin, cisplatin and etoposide), GSDME is specifically cleaved by caspase-3 at Asp^270^ within its linker, releasing the pore-forming domain (GSDME N-terminal domain) for plasma membrane disruption and thereby inducing pyroptosis (Fig. 3A). We treated A549 cells with the indicated concentrations of chemotherapeutic agent doxorubicin (0, 1, 2, 4, 8 μM) for 24 h and examined the N-terminal domain of GSDME (GSDME-N) by Western blot analysis, revealing an ~ 35-kDa cleavage fragment (Fig. 3B). Considering that EGFR is an upstream regulator of ERK1/2, we assumed that GSDME regulated the phosphorylation of ERK1/2 through EGFR. It was predicted that EGFR may interact with GSDME [35]. We explored potential interactors of GSDME in the BioGRID database (https://thebiogrid.org/) and found that EGFR is a candidate interactor of GSDME (Fig. 3C). Therefore, we examined whether GSDME physically interacted with EGFR. We constructed pcDNA3.1-FLAG-GSDME-HA (wild type), pcDNA3.1-FLAG-GSDME (D270A)-HA (GSDME (D270A) mutation) and pcDNA3.1-FLAG-GSDME-N plasmids and performed co-IP assays in HEK293FT and H1299 cells. We found that all three forms of GSDME (WT GSDME, GSDME (D270A) and GSDME-N) interacted with EGFR (Fig. 3D–F). EGFR consists of the extracellular and transmembrane fragment (ETTM), cytoplasmic and transmembrane fragment (CTTM) and the cytoplasmic fragment (CT) (Fig. 3G). Further analysis revealed that both the CTTM and CT of EGFR interact with GSDME, while the ETTM of EGFR could not interact with GSDME (Fig. 3H). To understand the interaction mechanism between GSDME and EGFR, we performed molecular docking using GSDME and the cytoplasmic domain of EGFR. As shown in Fig. 3I, there were two major interaction regions between GSDME and EGFR. Specifically, the hydrogen bonding pairs Y801_EGFR_-K98_GSDME_, Y813_EGFR_-T94_GSDME_, T993_EGFR_-R461_GSDME_, T993_EGFR_-K463_GSDME_, H998_EGFR_-L96_GSDME_ and H998_EGFR_-K463_GSDME_ played a key role in the binding of GSDME to EGFR; the hydrogen bonds between K737_EGFR_-E178_GSDME_ and K739_EGFR_-D204_GSDME_ also contributed to the association of both proteins.

### GSDME increases protein levels of EGFR and promotes phosphorylation of ERK1/2 in NSCLC cells

**Fig. 4:**
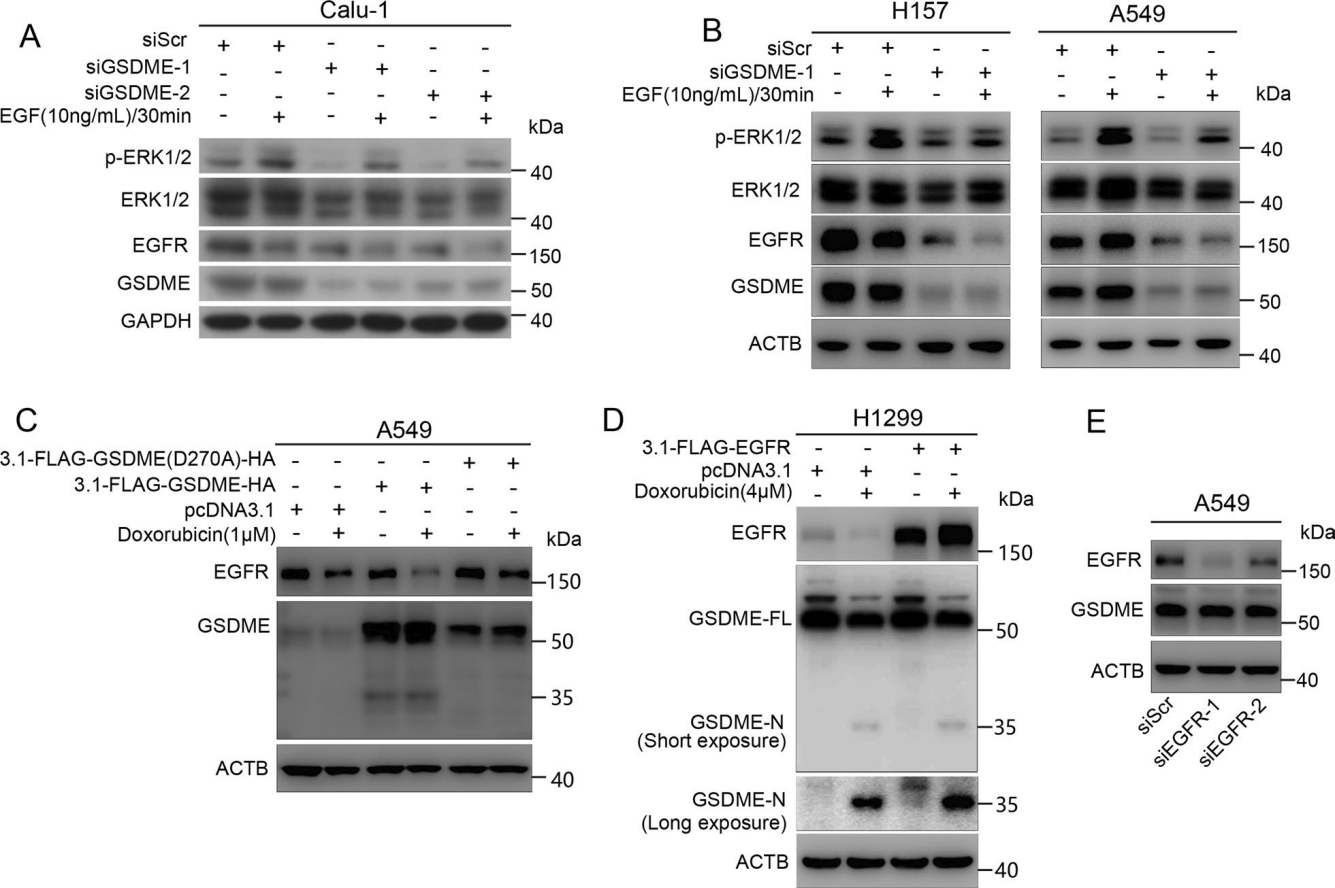
GSDME knockdown decreases the protein levels of EGFR and suppresses the phosphorylation of ERK1/2 in NSCLC cells. **A**, **B** Western blot analysis of EGFR, phosphorylated ERK1/2, ERK1/2 and GSDME levels in Calu-1, H157 and A549 cells treated with 10 ng/mL EGF for 30 min after transfection with siScr or siGSDME for 48 h. **C** Western blot analysis of EGFR and GSDME levels in A549 cells transfected with pcDNA3.1, pcDNA3.1-FLAG-GSDME-HA, or pcDNA3.1-FLAG-GSDME (D270A)-HA treated with or without 1 μM doxorubicin for 24 h. **D** Western blot analysis of EGFR and GSDME levels in H1299 cells transfected with pcDNA3.1 and pcDNA3.1-FLAG-EGFR treated with or without 4 μM doxorubicin for 24 h. **E** Western blot analysis of EGFR and GSDME in EGFR-depleted A549 cells. GAPDH or β-Actin was used as the loading control

Our data revealed that GSDME promotes cell proliferation and interacts with the CT of EGFR. We next investigated whether GSDME promotes the phosphorylation of ERK1/2 through EGFR. We examined the protein levels of EGFR and phosphorylated ERK1/2 in GSDME knockdown cells by Western blot analysis. We found that both EGFR and phosphorylated ERK1/2 levels decreased in GSDME knockdown Calu-1, H157 and A549 cells (Fig. 4A, B). Our previous work found that doxorubicin induces EGFR downregulation in a dose- and time-dependent manner in NSCLC cells [36]. Therefore, we treated GSDME-knockdown A549 cells with 2 μM doxorubicin for 24 h. As shown in Additional file 1: Fig. S3A, EGFR levels in GSDME-knockdown A549 cells were decreased after treatment with doxorubicin. In addition, we found that depletion of GSDME reduced EGFR protein levels (Fig. 4A, B, Additional file 1: Fig. S3B). When cells were treated with doxorubicin, overexpression of GSDME (D270A) versus GSDME-WT increased EGFR protein levels (Fig. 4C). To determine whether EGFR regulates GSDME, we knocked down or overexpressed EGFR in NSCLC cells (A549 and H1299, respectively) and examined the protein level of GSDME. As shown in Fig. 4 D and E, EGFR had no effect on GSDME protein levels. These data indicated that GSDME knockdown decreased the stability of EGFR, suggesting that GSDME might be a potent regulator of EGFR.

### GSDME knockdown enhances the degradation of EGFR

**Fig. 5:**
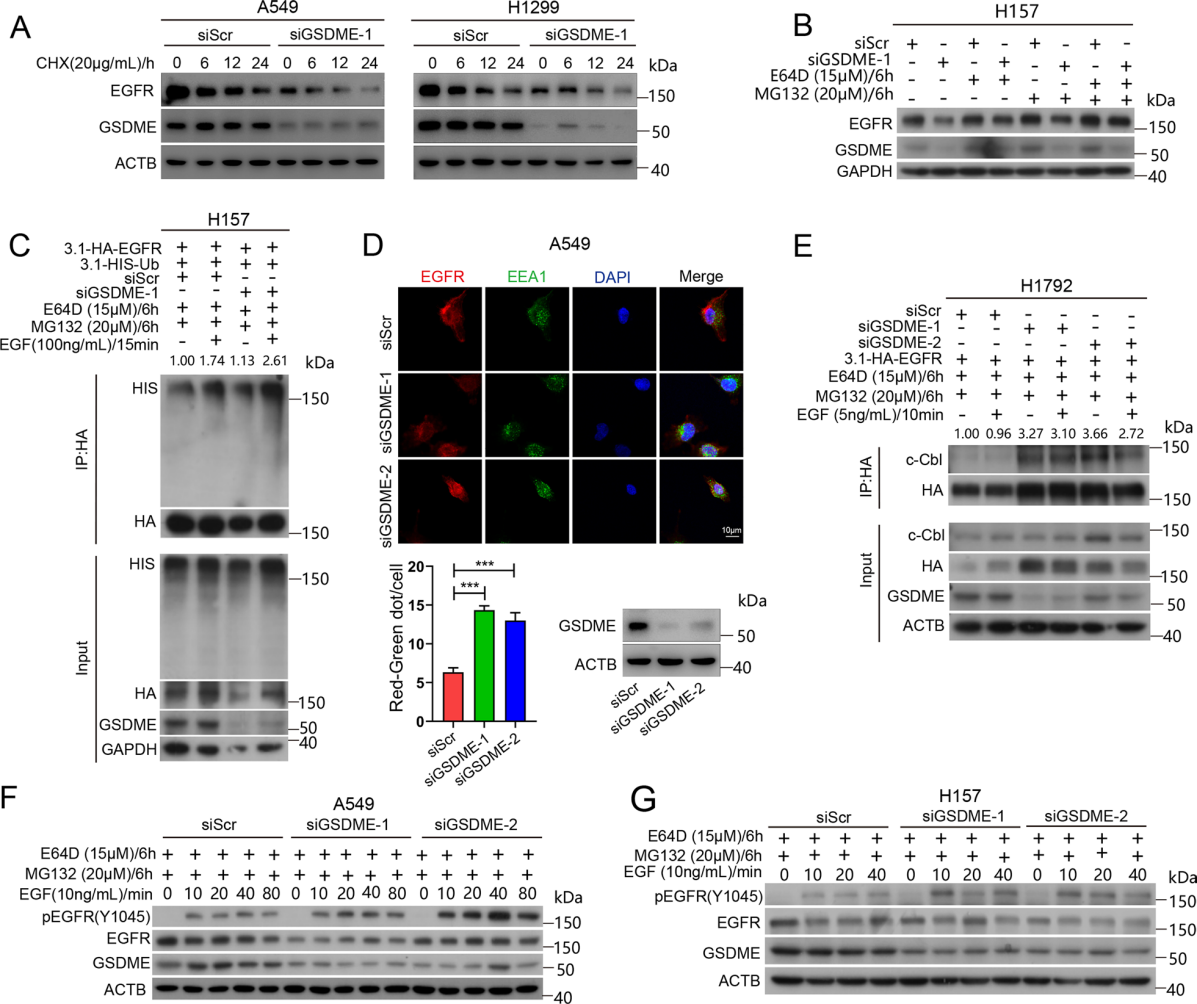
GSDME knockdown promotes the degradation of EGFR. **A** Western blot analysis of EGFR and GSDME levels in A549 and H1299 cells treated with 20 μg/mL cycloheximide (CHX) for 0 h, 6 h, 12 h and 24 h after transfection with siScr or siGSDME for 24 h. β-Actin was used as the loading control. **B** Western blot analysis of EGFR and GSDME levels in H157 cells treated with 15 μM E64D or 20 μM MG132 for 6 h after transfection with siScr or siGSDME for 24 h. GAPDH was used as the loading control. **C** EGFR ubiquitination was detected by a co-IP assay in GSDME-depleted H157 cells. H157 cells were transfected with pcDNA3.1-HA-EGFR, pcDNA3.1-HIS-Ub, siScr or siGSDME for 24 h and then treated with 15 μM E64D, 20 μM MG132 for 6 h and 100 ng/mL EGF for 15 min. A co-IP assay was performed with HA antibody, and the coeluted proteins were detected by Western blot analysis with HIS, HA and GSDME antibodies. The numbers shown above the bands were fold changes of band intensities relative to the control. The band intensities of HIS were quantified by Image J software and normalized to HA. The band intensity of HIS was relatively increased in GSDME-knockdown H157 cells. GAPDH was used as the loading control for the input. **D** Up: Immunofluorescence of EGFR (red fluorescence) and EEA1 (green fluorescence) in GSDME-depleted A549 cells. Scale bar, 10 μm. Down: quantification of the co-localization of EEA1 and EGFR. RNAi effency of A549 cells were measured by Western blot analysis. β-Actin was used as the loading control. **E** The interaction of EGFR and c-Cbl was detected by a co-IP assay in GSDME-depleted H1792 cells. Cells were transfected with pcDNA3.1-HA-EGFR, siScr or siGSDME for 24 h and then treated with 15 μM E64D, 20 μM MG132 for 6 h and 5 ng/mL EGF for 10 min. A Co-IP assay was performed with HA antibody, and the coeluted proteins were detected by Western blot analysis with c-Cbl, HA and GSDME antibodies. The numbers shown above the bands were fold changes of band intensities relative to the control. The band intensity of c-Cbl was quantified by Image J software and normalized to HA. c-Cbl level was relatively increased in GSDME knockdown H1792 cells. β-Actin was used as the loading control of the input. **F** Western blot analysis of phosphorylated EGFR (Y1045), EGFR and GSDME levels in A549 cells treated with 15 μM E64D or 20 μM MG132 for 6 h and 10 ng/mL EGF for 0 min, 10 min, 20 min, 40 min and 80 min after transfection with siScr or siGSDME for 24 h. **G** Western blot analysis of phosphorylated EGFR (Y1045), EGFR and GSDME levels in H157 cells treated with 15 μM E64D or 20 μM MG132 for 6 h and 10 ng/mL EGF for 0 min, 10 min, 20 min and 40 min after transfection with siScr or siGSDME for 24 h. β-Actin was used as the loading control

To examine whether GSDME affects the stability of EGFR, we treated GSDME-knockdown A549 and H1299 cells with cycloheximide (CHX), an inhibitor of protein synthesis. Our data revealed that GSDME knockdown decreased the stability of EGFR (Fig. 5A). To further investigate the pattern of EGFR degradation in NSCLC cells, we treated GSDME-knockdown H157 cells with aloxistatin (E64D, a cell-permeable and irreversible broad-spectrum cysteine protease inhibitor) and MG132 (a proteasome inhibitor). Both E64D and MG132 rescued the degradation of EGFR induced by GSDME knockdown, and the two in combination further suppressed the degradation of EGFR, indicating that GSDME knockdown induced EGFR degradation through lysosomal and proteasoml pathway (Fig. 5B). GSDME knockdown increased the ubiquitination of EGFR (Fig. 5C). We examined the stages of EGFR endocytosis in GSDME-depleted A549 cells. Immunofluorescence staining of EGFR and EEA1 revealed that GSDME knockdown promoted the colocalization of EGFR and EEA1, indicating that GSDME knockdown promoted the formation of early endosome-containing EGFR on the plasma membrane (Fig. 5D). c-Cbl is an E3 ubiquitin ligase that induces monoubiquitination and lysosome-mediated degradation of EGFR [8, 37]. To test whether GSDME knockdown promotes EGFR ubiquitination and degradation by c-Cbl. We overexpressed EGFR in GSDME-depleted H1792 cells, and co-IP assays indicated that GSDME knockdown enhanced the interaction of c-Cbl and EGFR (Fig. 5E). Phosphorylation of EGFR^Y1045^ acts as the degron of EGFR and is recognized by c-Cbl, enhancing the ubiquitination and degradation of EGFR. EGFR stability is tightly regulated upon EGF stimulation, and the EGFR ubiquitination threshold is controlled by EGF concentration [10]. We knocked down GSDME in A549 and H157 cells and added EGF for the indicated times (0, 10, 20, 40, 80 min) to induce EGFR^Y1045^ phosphorylation. Western blot analysis revealed that EGFR^Y1045^ phosphorylation increased in GSDME-depleted cells (Fig. 5F, G). We overexpressed GSDME WT and GSDME (D270A) in H1299 cells and added EGF to induce EGFR^Y1045^ phosphorylation. Western blot analysis revealed that overexpression of GSDME (D270A) reduced EGFR^Y1045^ phosphorylation (Additional file 1: Fig. S4). These results indicated that GSDME knockdown promotes the degradation of EGFR.

### The GSDME N-terminal fragment promotes EGFR degradation

**Fig. 6:**
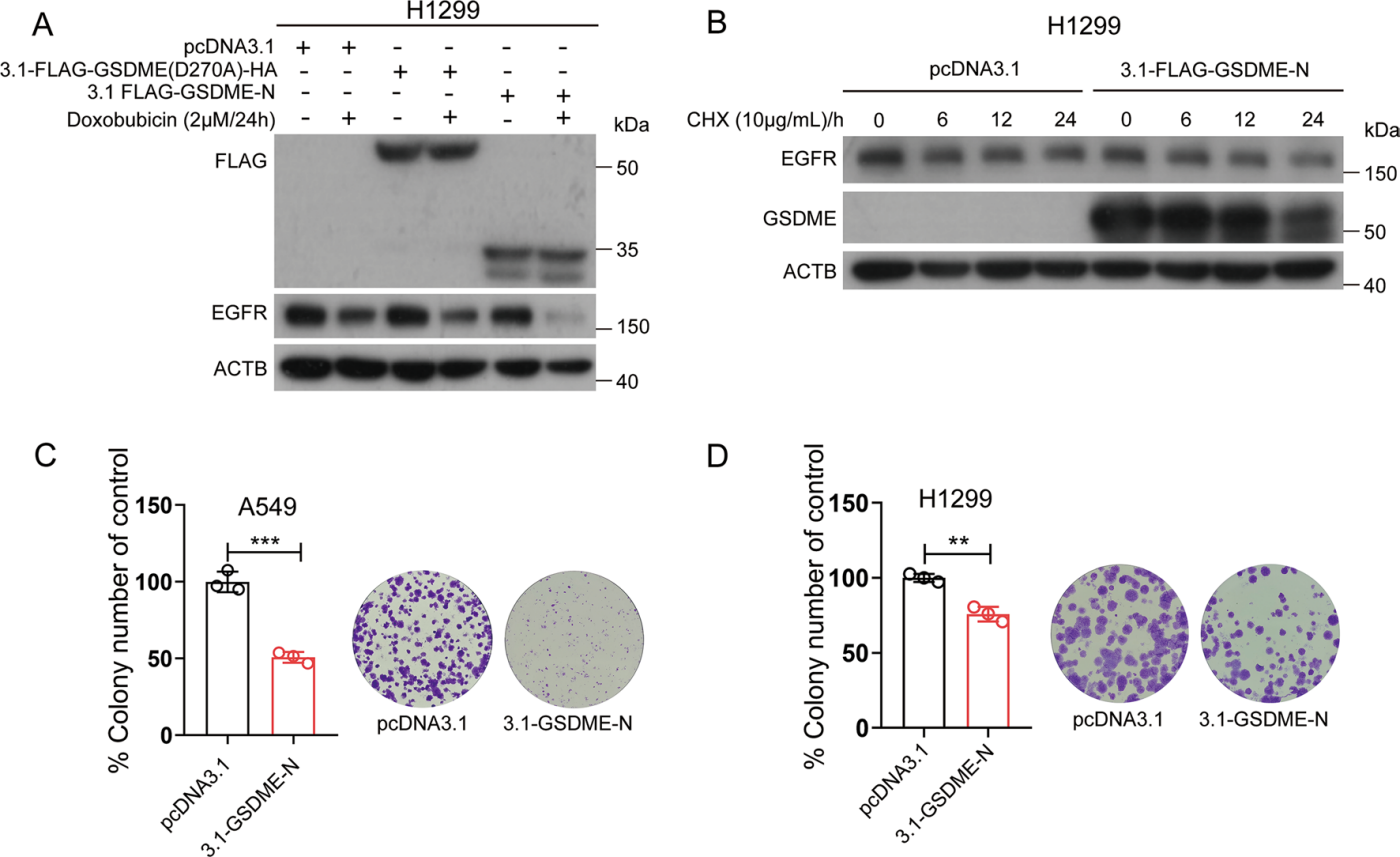
The GSDME-N fragment promotes EGFR degradation. **A** Western blot analysis of FLAG and EGFR levels in H1299 cells. H1299 cells were transfected with pcDNA3.1, pcDNA3.1-FLAG-GSDME (D270A)-HA, pcDNA3.1-FLAG-GSDME-N and treated with or without 2 μM doxorubicin for 24 h. **B** Western blot analysis of EGFR and GSDME levels in H1299 cells treated with 10 μg/mL cycloheximide (CHX) for 0 h, 6 h, 12 h, and 24 h after transfection with pcDNA3.1 or pcDNA3.1-FLAG-GSDME-N for 24 h. **C**, **D** Colony formation efficiency of A549 and H1299 cells after transfection with pcDNA3.1 or pcDNA 3.1-GSDME-N plasmids for 14 days. Data are presented as the mean ± SD and are representative of three independent experiments. The significant differences between the two groups were analyzed by two-sided unpaired Student’s *t* tests (**p < 0.01, ***p < 0.001)

GSDME adopts a two-domain architecture (GSDME-N and GSDME-C), and the two domains are capable of binding to each other and inhibiting the function of GSDME-N. When cells were treated with doxorubicin, caspase-3 was activated, cleaved GSDME at Asp^270^ and released GSDME-N. We overexpressed GSDME (D270A) and GSDME-N in H1299 cells and then treated them with doxorubicin for 24 h to downregulate EGFR. Western blot analysis revealed that GSDME-N promoted the downregulation of EGFR (Fig. 6A). Next, we overexpressed GSDME-N in H1299 cells and treated them with CHX for the indicated times (0, 6, 12, and 24 h). As shown in Fig. 6B, overexpression of GSDME-N reduced the stability of EGFR. The colony formation ability decreased in GSDME-N overexpression cells (Fig. 6C, D). In general, GSDME-FL increased the stability of EGFR, while GSDME-N induced EGFR degradation.

**GSDME knockdown inhibits EGFR**^**Y1173**^ **phosphorylation**.

**Fig. 7:**
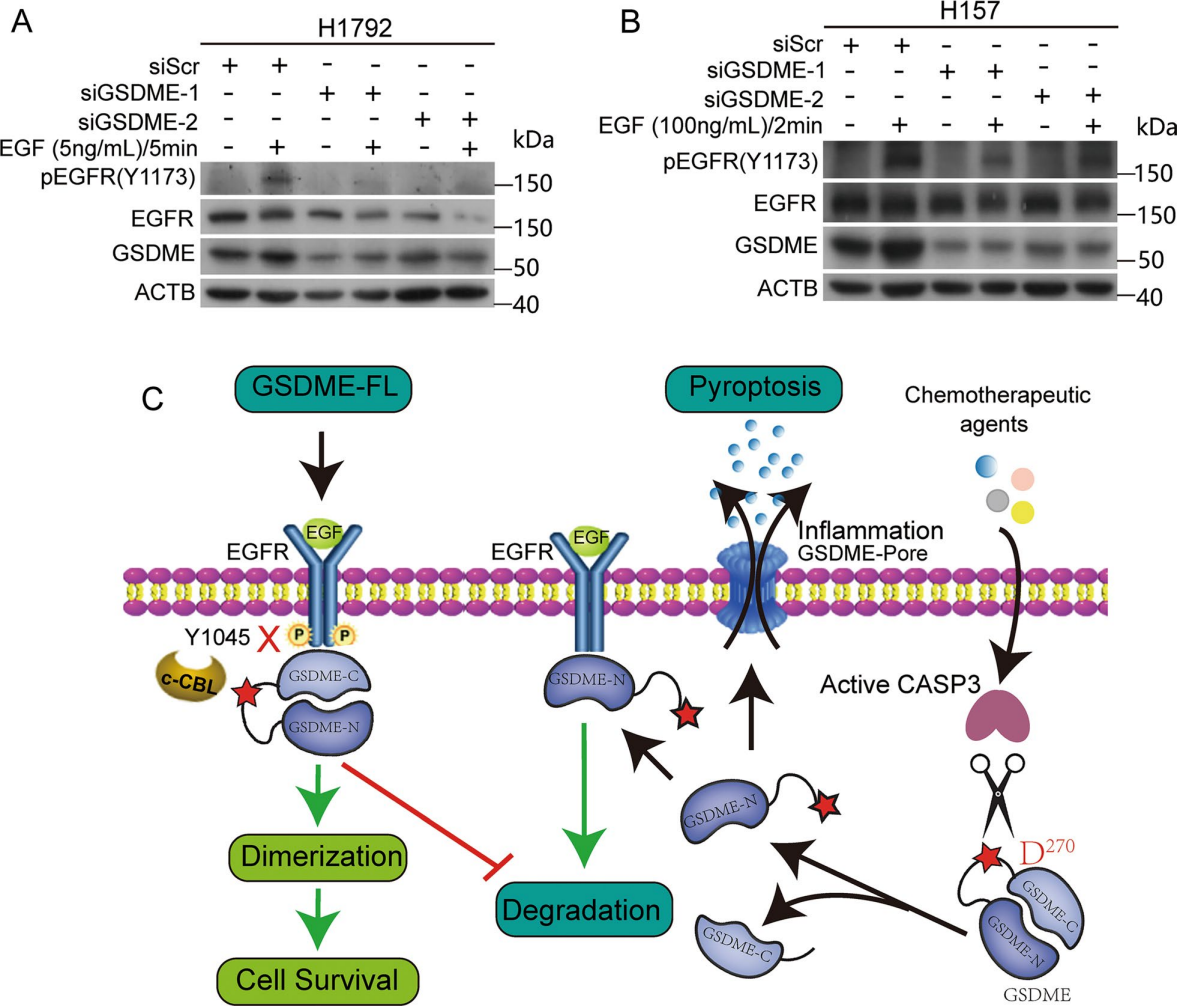
GSDME knockdown inhibits EGFR^Y1173^ phosphorylation. **A**, **B** Western blot analysis of phosphorylated EGFR (Y1173), EGFR and GSDME levels in GSDME-depleted H1792 and H157 cells treated with or without 5 ng/mL EGF/5 min (H1792) and 100 ng/mL EGF/2 min (H157), respectively. β-Actin was used as the loading control. **C** Working model for regulation of GSDME and EGFR. Full-length GSDME (GSDME-FL) promotes cell proliferation by enhancing and maintaining EGFR signaling. GSDME-FL enhances EGFR signaling by promoting EGFR dimerization. GSDME-FL maintains EGFR signaling by physically interacting with EGFR and covering the EGFR^Y1045^ site, which contributes to its stabilization. When cells are treated with chemotherapeutic agents (such as doxorubicin), caspase-3 cleaves GSDME at Asp^270^ and releases the GSDME N-terminal fragment. Cleavage allows the GSDME pore-forming domain (GSDME-N) to perforate the plasma membrane and induce pyroptosis. Moreover, GSDME-N binds to EGFR on the membrane and promotes EGFR degradation

Our previous data revealed that GSDME affected the activity of EGFR by regulating the levels of EGFR, further promoting cell proliferation. We next examined whether GSDME regulated EGFR dimerization. Co-IP assays indicated that GSDME knockdown inhibited EGFR dimerization (Additional file 1: Fig. S5). We assumed that when interacting with the CT of EGFR, GSDME promotes EGFR dimerization and cell proliferation. As phosphorylation of EGFR^Y1173^ indicates an active EGFR cellular signaling pathway, we examined the EGFR^Y1173^ phosphorylation level in GSDME-depleted cells. As shown in Fig. 7A, B, GSDME knockdown reduced the phosphorylation of EGFR^Y1173^ in H1792 and H157cells. These results indicated that GSDME knockdown suppressed the activity of the EGFR downstream signaling pathway by inhibiting EGFR dimerization.

## Discussion

GSDME was recently identified as a promoter of pyroptosis owing to its cleavage by caspase-3 [17, 38]. In addition to its necrotic activity, GSDME has been proposed to possess tumor suppressor activity in gastric, breast and colorectal cancer. GSDME is generally downregulated in tumor tissues and cells [18, 39]. The expression of GSDME suppresses the colony formation and proliferation of cancer cells [19, 22, 40]. GSDME has been recently declared to inhibit tumor progression in breast, gastric and colorectal cancer by activating antitumor immunity [22]. However, little is known about the role of GSDME in NSCLC cells, and the underlying mechanisms remain to be fully determined. Does GSDME simply function to induce pyroptosis and thereby inhibit cell survival and proliferation? Does GSDME expression have consequences beyond pyroptosis? Here, we showed that GSDME is upregulated in LUSC and LUAD (Fig. 1A, B) and expressed in various NSCLC cell lines (A549, Calu-1, H1299 and H157) (Fig. 1C). These results were different from previous observations that GSDME expression was negative or low in other cancer cells [18–20]. The upregulation of GSDME in tumors compared with normal tissues might be a result of intrinsic genetic/epigenetic alterations in tumors or a result of the tumorigenic process outcome (such as increased cell death induced by hypoxia) [41]. We also found that GSDME knockdown reduced the proliferation of NSCLC cancer cells in vitro (Fig. 1D and E, Additional file 1: Fig. S1). Mechanistically, both GSDME-FL and GSDME-N physically interact with EGFR, a very important membrane receptor that plays a critical role in cell survival and proliferation. Molecular docking analysis showed that 6 hydrogen bonding pairs play a key role in the binding of GSDME to EGFR (Fig. 3I). GSDME knockdown inhibited EGFR dimerization and phosphorylation at tyrosine 1173 (EGFR^Y1173^), which activated ERK1/2 (Fig. 7) and promoted EGFR degradation via phosphorylation at tyrosine 1045 (EGFR^Y1045^) (Fig. 5). Moreover, GSDME-FL increased the stability of EGFR, while the GSDME-N fragment induced EGFR degradation. Therefore, in addition to directly promoting pyroptosis, the cytotoxic effect of GSDME depletion can be further amplified by the EGFR-ERK1/2 cascade, which affects cell survival and proliferation.

Many studies have reported that the expression of GSDME in tumors is suppressed by methylation and functions as a tumor suppressor in various tumors. A recent study have reported that GSDME is ubiquitously expressed in lung cancer [31] and that the expression of GSDME in OSCC (oral squamous cell carcinoma) is significantly higher than that in normal mucosa [41]. It was also reported that the protein levels of GSDME were significantly increased in the colonic mucosa of IBD patients compared to healthy controls and that GSDME increases the release of HMGB1 (one of the DAMPs that has been recognized) from intestinal epithelial cells (IECs), which contributes to CAC tumorigenesis [23]. These findings suggest that GSDME might play a dual role in tumor progression.

The GSDME protein consists of conserved N- and C-terminal globular domains, which are separated by a flexible hinge region. The GSDME N-terminal domain was shown to execute inflammatory cell death via pore formation. This function is apparently inhibited by the C-terminal domain (GSDME-C) in the full-length protein. Expression of the GSDME-N domain is highly toxic to *Escherichia coli*, whereas little cytotoxicity is observed with GSDME-FL and GSDME-C domains [29, 42]. GSDME could be specifically cleaved by caspase-3 at Asp^270^ when cells were treated with chemotherapy drugs (such as doxorubicin, cisplatin and etoposide), generating GSDME-N (the pore-forming domain), which can target the cell membrane, leading to swelling and cell death [38]. Our data reveal that GSDME-FL, GSDME (D270A) and GSDME-N all physically interact with EGFR (Fig. 3). However, in contrast to GSDME-FL, which stabilizes EGFR, the GSDME-N fragment promotes the degradation of EGFR (Fig. 6). We speculated that GSDME-FL covers the EGFR ^Y1045^ site, increasing its stability and causing the abnormal activation of EGFR, while the GSDME-N fragment binds to EGFR on the membrane and promotes its degradation (Fig. 7C). The function of the GSDME-N fragment might be related to its pore-forming function, and further studies are needed to investigate the role of the GSDME-N fragment. We propose that GSDME-FL supports cell proliferation when cells are not exposed to drug treatment. However, chemotherapeutic agents exposure induce the cleavage of GSDME, and GSDME-N translocates to the membrane to form pores to induce pyroptosis, releasing EGFR for degradation. Our findings characterize the distinct effects of GSDME-FL and GSDME-N on EGFR stability and degradation.

Taken together, our work indicates that GSDME-mediated EGFR stabilization contributes to the development of NSCLC by activating the ERK1/2 pathway. In addition to its role in pyroptosis, GSDME directly interacts with EGFR and contributes to its stability. GSDME-FL increases EGFR stability and help cells against pyroptosis under chemotherapeutic agents treatment. This maybe a synergistically protective mechanism for these cells. Our findings highlight the emerging role of GSDME-mediated EGFR stability in promoting the proliferation of NSCLC cells and provide new insights for the future development of NSCLC therapeutic strategies by targeting GSDME-mediated EGFR stability. Although the specific regulatory mechanism of GSDME and EGFR requires more study, our findings indicate that the GSDME-EGFR interaction may be a ubiquitous cancer-promoting factor. Finally, further studies are warranted to investigate the role of GSDME in the development of NSCLC.

## Conclusions

In summary, our results indicate that GSDME maintains EGFR signaling and promotes cell survival by enhancing the dimerization and activation of EGFR. Moreover, GSDME physically interacts with EGFR, covering the EGFR^Y1045^ site and contributing to its stability. GSDME-FL increased the stability of EGFR, while the GSDME N-terminal fragment induced EGFR degradation. Therefore, the GSDME-EGFR interaction plays an important role in NSCLC development, reveal a previously unrecognized link between GSDME and EGFR stability and offer new insight into cancer pathogenesis.

## Supplementary Information

**Additional file 1.** **Figure S1.** GSDME depletion suppresses the proliferation of NSCLC cells in vitro. **Figure S2.** The 3D structures of GSDME and EGFR. **Figure S3.** GSDME knockdown decreases protein levels of EGFR. **Figure S4.** Overexpression of GSDME (D270A) decreases phosphorylation of EGFR^Y1045^. **Figure S5.** GSDME knockdown inhibited EGFR dimerization. **Figure S6.** Plasmid overexpression efficiency of GSDME-FL, GSDME-N and GSDME (D270A). **Figure S7.** An example figure of the flow cytometry gating strategy. Abbreviations of TCGA Cancers. KEY RESOURCES TABLE.

## Data Availability

All data generated or analyzed during this study are included in this published article and its Additional file 1: information files. The datasets analyzed to support the conclusion of article are available from the corresponding author upon reasonable request.

## Abbreviations

CASP3: Caspase-3
c-Cbl: C-Cbl-proto-oncogene
CCND1: Cyclin D1
Co-IP: Coimmunoprecipitation
CT: Cytoplasmic fragment
CTTM: Cytoplasmic and transmembrane fragment
DAMPs: Damage-associated molecular patterns
EGF: Epidermal growth factor
EGFR: Epidermal growth factor receptor
ERK: Ras–extracellular signal-regulated kinase
ETTM: Extracellular and transmembrane fragment
GSDME: Gasdermin E
LUAD: Lung adenocarcinoma
LUSC: Lung squamous cell carcinoma
NSCLC: Non-small cell lung cancer
TKIs: Tyrosine kinase inhibitors
