# Supplementary material for: Gasdermin E regulates the stability and activation of EGFR in human non-small cell lung cancer cells
Source: Cell Commun Signal. 2023 Apr 21;21:83. doi: 10.1186/s12964-023-01083-7 (PMC10120120; doi:10.1186/s12964-023-01083-7)
Supplement: Supplementary file 2 — Additional file 1. Figure S1. GSDME depletion suppresses the proliferation of NSCLC cells in vitro. Figure S2. The 3D structures of GSDME and EGFR. Figure S3. GSDME knockdown decreases protein levels of EGFR. Figure S4. Overexpression of GSDME (D270A) decreases phosphorylation of EGFRY1045. Figure S5. GSDME knockdown inhibited EGFR dimerization. Figure S6. Plasmid overexpression efficiency of GSDME-FL, GSDME-N and GSDME (D270A). Figure S7. An example figure of the flow cytometry gating strategy. Abbreviations of TCGA Cancers. KEY RESOURCES TABLE. [file 12964_2023_1083_MOESM2_ESM.docx]

**Supplementary information**

**Gasdermin E regulates the stability and activation of EGFR in human non-small cell lung cancer cells**

Limei Xu^1^, Feifei Shi^1^, Yingdi Wu^1^, Shun Yao^1^, Yingying Wang^1^, Xukai Jiang^2^, Ling Su^1,^* and Xiangguo Liu^1,^*

^1^Shandong Provincial Key Laboratory of Animal Cell and Developmental Biology, School of Life Sciences, Shandong University, Qingdao, China.

^2^ National Glycoengineering Research Center, Shandong University, Qingdao, China.

**Running title:** GSDME regulates EGFR stability.

**Supplementary Figure 1.** **GSDME depletion suppresses the proliferation of NSCLC cells in vitro.**

**
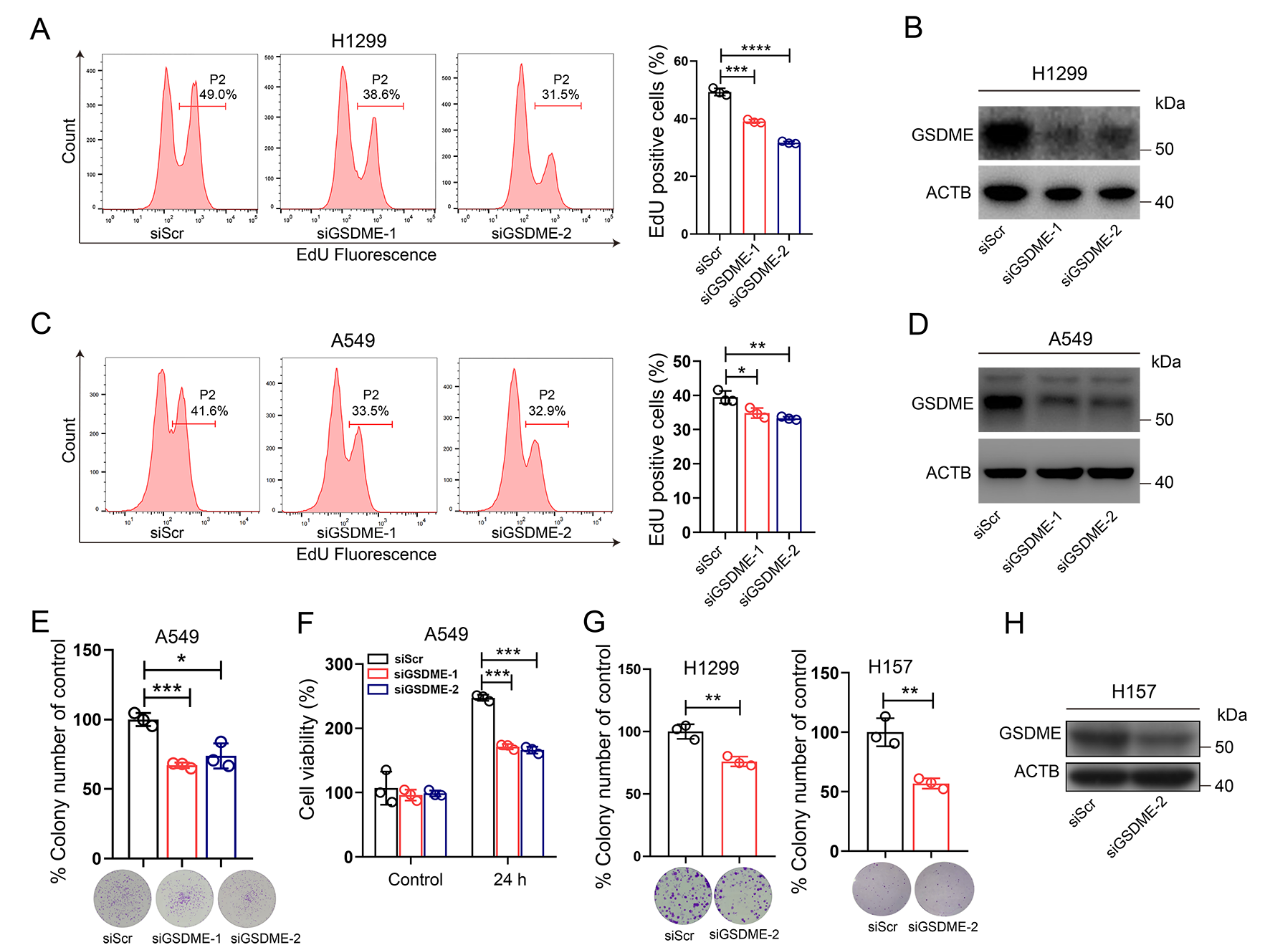
**

**A** and **C** EdU incorporation was measured by flow cytometry in H1299 and A549 cells transfected with siScr or siGSDME for 48 or 24 h, respectively. **B** and **D** The RNAi efficiency of Calu-1 cells was measured by Western blot analysis. β-Actin was used as the loading control. **E** Colony formation efficiency of A549 cells after transfection with scrambled siRNA (siScr) or siRNA targeting GSDME (siGSDME) for 14 days. **F** Cell viability was measured by CCK-8 assay. A549 cells were transfected with siScr, siGSDME-1 and siGSDME-2. **G** Colony-forming efficiency of H1299 and H157 cells after transfection with siScr or siGSDME for two weeks. **H** The RNAi efficiency of H157 cells was measured by Western blot analysis. β-Actin was used as the loading control. Data are presented as the mean ± SD and are representative of three independent experiments. The statistical differences between the two groups were analyzed by two-sided unpaired Student’s *t test*s (**p* < 0.05, ***p* < 0.01, ****p* < 0.001, *****p* < 0.0001).

**Supplementary Figure 2. The 3D structures of GSDME and EGFR.**

**
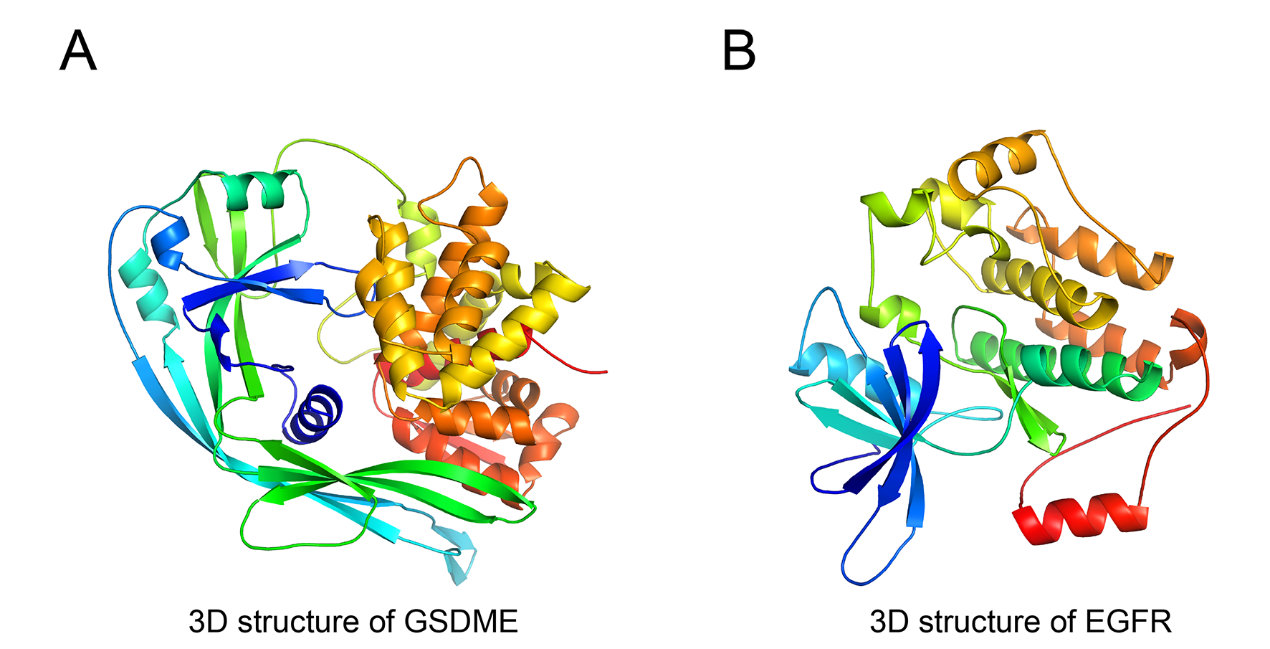
**

**A** The 3D structure of GSDME predicted through the AlphaFold2 method. **B** The 3D structure of EGFR from the RCSB Protein Data Bank (PDB code: 4RJ4).

**Supplementary Figure 3. GSDME knockdown decreases protein levels of EGFR.**





**A** Western blot analysis of EGFR and GSDME levels in A549 cells treated with 2 μM doxorubicin for 24 h after transfection with siScr or siGSDME for 24 h. **B** Western blot analysis of EGFR and GSDME in GSDME-depleted Calu-1, H1299 and H157 cells. β-Actin was used as the loading control.

**Supplementary Figure 4. Overexpression of GSDME (D270A) decreases phosphorylation of EGFR^1045^.**


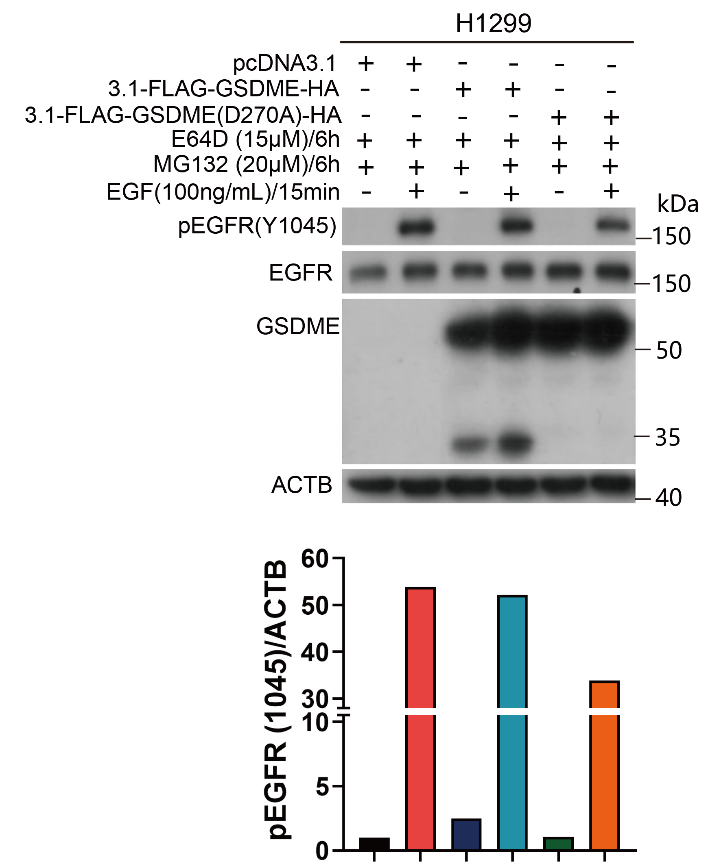


Western blot analysis of phosphorylated EGFR (Y1045), EGFR and GSDME levels in H1299 cells transfected with pcDNA3.1, pcDNA3.1-FLAG-GSDME-HA, pcDNA3.1-FLAG-GSDME (D270A)-HA treated with 15 μM E64D and 20 μM MG-132 for 6 h and 100 ng/mL EGF for 15 min. β-Actin was used as the loading control. The band intensities were quantified by Image J software and normalized to β-Actin.

**Supplementary Figure 5. GSDME knockdown inhibited EGFR dimerization.**

**
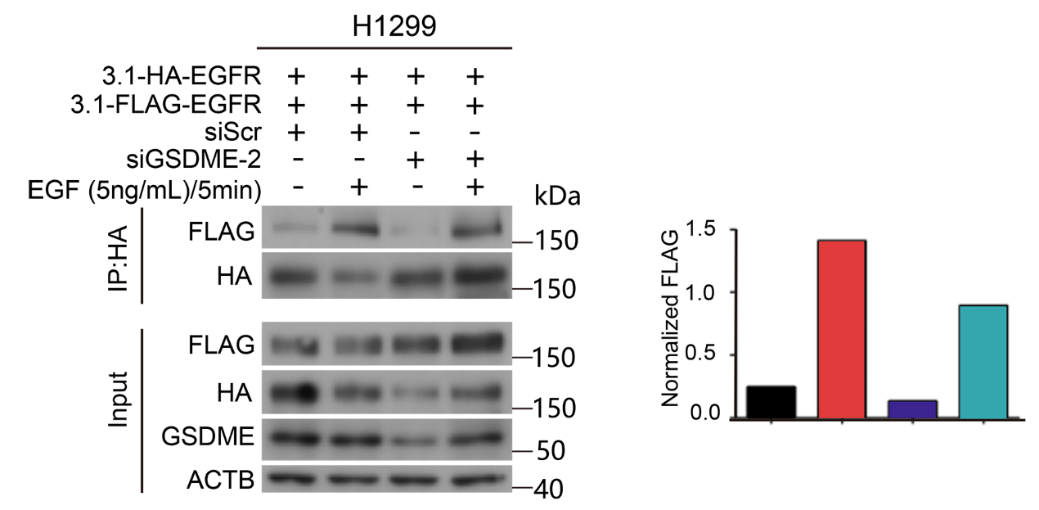
**

Left: EGFR dimerization was detected by a co-IP assay in GSDME-depleted H1299 cells. H1299 cells were transfected with pcDNA3.1-HA-EGFR, pcDNA3.1-FLAG-EGFR, siScr or siGSDME for 24 h and then treated with 5 ng/mL EGF for 5 min. A co-IP assay was performed with HA antibody, and the coeluted proteins were detected by Western blot analysis with FLAG, HA and GSDME antibodies. β-Actin was used as the loading control of the input. Right: Relative quantitative protein expression of FLAG. The band intensities were quantified by Image J software and normalized to FLAG.

**Supplementary Figure 6.** **Plasmid overexpression efficiency of GSDME-FL, GSDME-N and GSDME (D270A).**


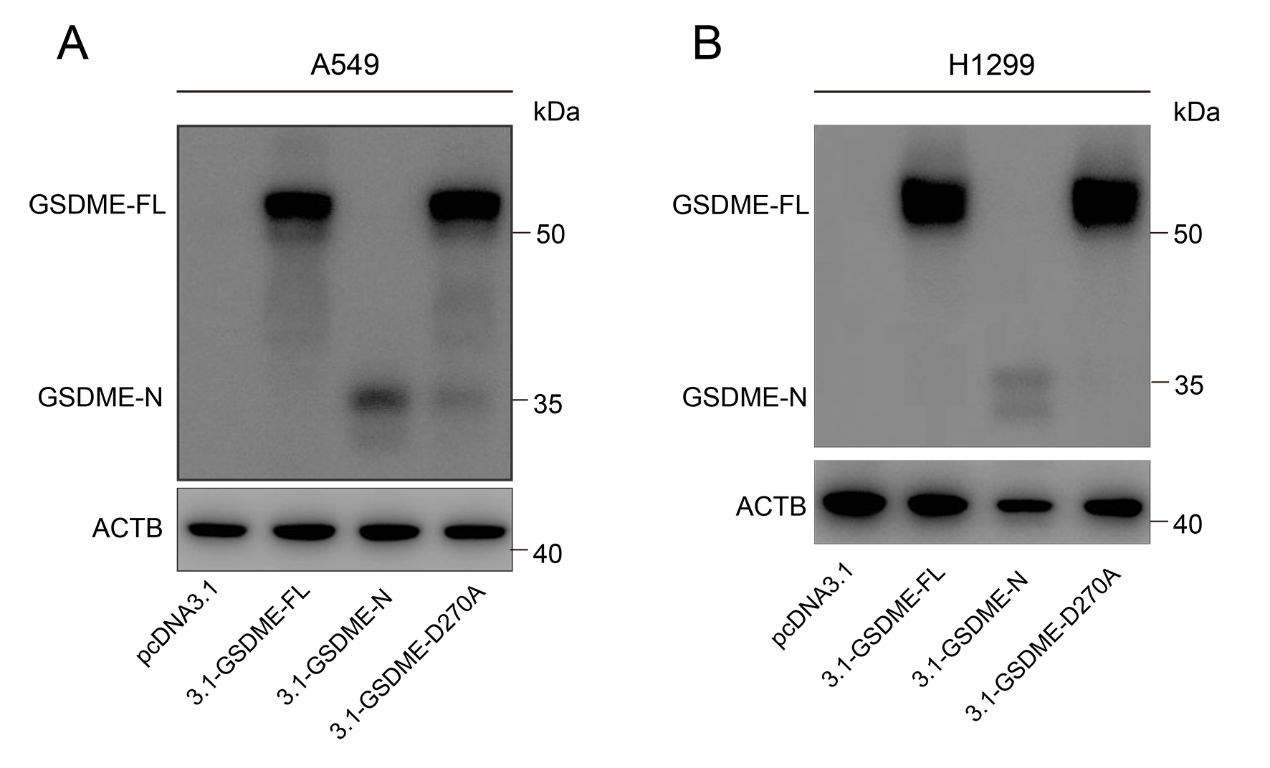


**A** and **B** Overexpression efficiency of pcDNA3.1-GSDME-FL, pcDNA3.1-GSDME-N and pcDNA3.1-GSDME (D270A) in A549 and H1299 cells was detected by Western blot analysis. The protein level of GSDME was measured after transfection with pcDNA3.1, pcDNA3.1-GSDME-FL, pcDNA3.1-N and pcDNA3.1-GSDME (D270A) for 24 h. β-Actin was used as the loading control.

**Supplementary Figure 7. An example figure of the flow cytometry gating strategy.**


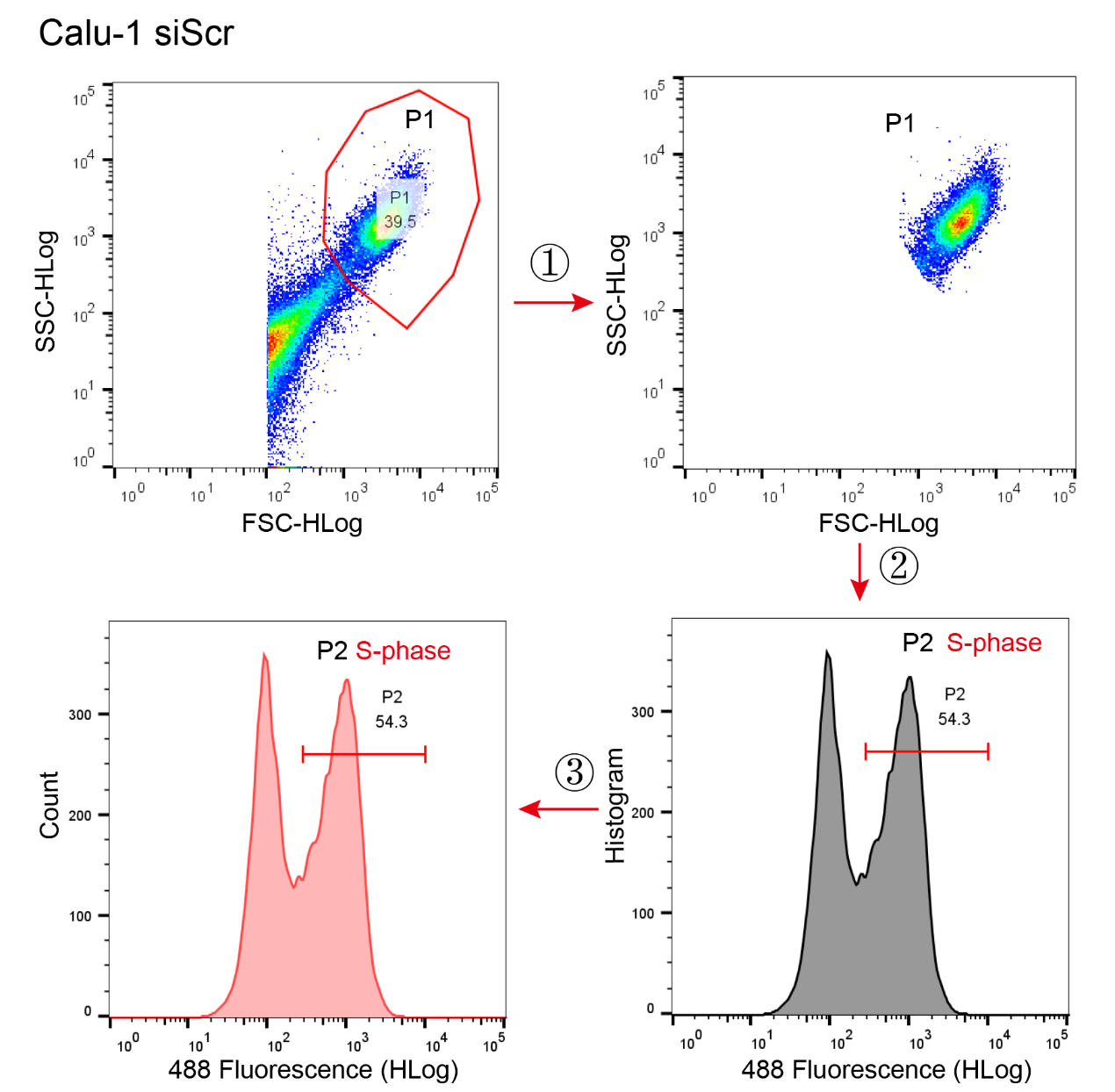


**Abbreviations of TCGA Cancers**

| **Cohort** | **Full name** |
| --- | --- |
| BLCA | Bladder Urothelial Carcinoma |
| BRCA | Breast invasive carcinoma |
| CESC | Cervical squamous cell carcinoma and endocervical adenocarcinoma |
| CHOL | Cholangiocarcinoma |
| COAD | Colon adenocarcinoma |
| ESCA | Esophageal carcinoma |
| GBM | Glioblastoma multiforme |
| HNSC | Head and Neck squamous cell carcinoma |
| KICH | Kidney Chromophobe |
| KIRC | Kidney renal clear cell carcinoma |
| KIRP | Kidney renal papillary cell carcinoma |
| LIHC | Liver hepatocellular carcinoma |
| LUAD | Lung adenocarcinoma |
| LUSC | Lung squamous cell carcinoma |
| PAAD | Pancreatic adenocarcinoma |
| PRAD | Prostate adenocarcinoma |
| PCPG | Pheochromocytoma and Paraganglioma |
| READ | Rectum adenocarcinoma |
| SARC | Sarcoma |
| SKCM | Skin Cutaneous Melanoma |
| THCA | Thyroid carcinoma |
| THYM | Thymoma |
| STAD | Stomach Adenocarcinoma |
| UCEC | Uterine Corpus Endometrial Carcinoma |

**KEY RESOURCES TABLE**

| REAGENT or RESOURCE | SOURCE | IDENTIFIER |
| --- | --- | --- |
| Antibodies |  |  |
| Rabbit anti-GSDME | Cell signaling Technology | Cat# 84005 |
| Rabbit anti p-ERK1/2 | Cell signaling Technology | Cat# 4370 |
| Rabbit anti ERK1/2 | Cell signaling Technology | Cat# 9102 |
| Mouse anti-CCND1 | Cell signaling Technology | Cat# 2926 |
| Rabbit anti-EGFR | Santa Cruz | Cat# sc-03 |
| Rabbit anti p-EGFR Y1045 | Invitrogen | Cat# PA5-17816 |
| Rabbit anti p-EGFR Y1173 | Invitrogen | Cat# MA5-15158 |
| Mouse anti-ACTB | Sigma-Aldrich | Cat# A1978 |
| Mouse anti-GAPDH | Sigma-Aldrich | Cat# G8795 |
| Mouse anti-FLAG | Sigma-Aldrich | Cat# F1804 |
| Rabbit anti-FLAG | Sigma-Aldrich | Cat# F7425 |
| Mouse anti-EEA1 | Sigma-Aldrich | Cat# E7659 |
| Rabbit anti-HIS | Sigma-Aldrich | Cat# SAB1306085 |
| Mouse anti-HA | Proteintech | Cat# 66006-1-IG |
| Rabbit anti-cCbl | Proteintech | Cat# 25818-1-AP |
| Chemicals |  |  |
| Doxorubicin | Selleck | Cat# E2516 |
| MG-132 | Selleck | Cat# S2619 |
| EGF | Sangon Biotech | Cat# C610033 |
| E64D | Medchem Express | Cat# HY-100229 |
| Cycloheximide | Medchem Express | Cat# HY-123 |
| Softare |  |  |
| ImageJ | National Institutes of Heath | RRID:SCR_003070 |
| Adobe IIIustrator CC | Adobe Systems | RRID:SCR_010279 |
| GraphPad Prism 8.0 | GraphPad Software | https://www.graphpad.com/scientific-software/prism/ |
| FlowJo_V10 | BD Biosciences | https://www.bdbiosciences.com/zh-cn/products/software/flowjo-v10-software |
| PyMOL | DeLano Scientific LLC | https://pymol.org/2/ |
| Other |  |  |
| jetPRIME | Polyplus | Cat#101000001 |
| LipoMAX | Sudgen Biotechnology | Cat# 32012 |
| EdU | Beyotime | Cat# C0071 |
| UALCAN analysis | This paper | http://ualcan.path.uab.edu/analysis.html |
| TCGA data | This paper | http://www.cbioportal.org/ |
| pcDNA3.1 | Invitrogen | Cat# V79020 |
| pcDNA3.1-FLAG-GSDME-HA | This paper | N/A |
| pcDNA3.1-GSDME-HA | This paper | N/A |
| pcDNA3.1-FLAG-GSDME  (D270A)-HA | This paper | N/A |
| pcDNA3.1-FLAG-GSDME-N | This paper | N/A |
| pcDNA3.1-FLAG-EGFR | This paper | N/A |
| pcDNA3.1-HA-EGFR | This paper | N/A |
| pcDNA3.1-FLAG-EGFR (ETTM) | This paper | N/A |
| pcDNA3.1-FLAG-EGFR(CTTM) | This paper | N/A |
| pcDNA3.1-FLAG-EGFR (CT) | This paper | N/A |
| pcDNA3.1-HIS-UB | This paper | N/A |
